# Harnessing slow event-related fMRI to investigate trial-level brain-behavior relationships during object identification

**DOI:** 10.3389/fnhum.2024.1506661

**Published:** 2024-11-12

**Authors:** Stephen J. Gotts, Adrian W. Gilmore, Alex Martin

**Affiliations:** ^1^Section on Cognitive Neuropsychology, Laboratory of Brain and Cognition, National Institute of Mental Health, National Institutes of Health, Bethesda, MD, United States; ^2^Department of Psychological and Brain Sciences, University of Delaware, Newark, DE, United States

**Keywords:** effect size, correlation, BOLD fMRI, test–retest reliability, response time

## Abstract

Understanding brain-behavior relationships is the core goal of cognitive neuroscience. However, these relationships—especially those related to complex cognitive and psychopathological behaviors—have recently been shown to suffer from very small effect sizes (0.1 or less), requiring potentially thousands of participants to yield robust findings. Here, we focus on a much more optimistic case utilizing task-based fMRI and a multi-echo acquisition with trial-level brain-behavior associations measured within participant. In a visual object identification task for which the behavioral measure is response time (RT), we show that while trial-level associations between BOLD and RT can similarly suffer from weak effect sizes, converting these associations to their corresponding group-level effects can yield robust peak effect sizes (Cohen’s *d* = 1.0 or larger). Multi-echo denoising (Multi-Echo ICA or ME-ICA) yields larger effects than optimally combined multi-echo with no denoising, which is in turn an improvement over standard single-echo acquisition. While estimating these brain-behavior relationships benefits from the inclusion of a large number of trials per participant, even a modest number of trials (20–30 or more) yields robust group-level effect sizes, with replicable effects obtainable with relatively standard sample sizes (*N* = 20–30 participants per sample).

## Introduction

A primary goal of neuroscience - and cognitive neuroscience in particular - is to understand the brain mechanisms that support behavior. Studies that attempt to empirically examine the relationship between brain and mind require direct examination of which sources of neural variability are actually related to behavioral variability in cognitive domains of interest. A common approach in task-based neuroimaging studies such as those using fMRI is to correlate the mean BOLD response in a certain task across participants with a behavioral measure taken from the same participants (for discussion, see Rousselet and Pernet, 2012; Vul et al., 2009; Yarkoni and Braver, 2010). A similar approach to examining inter-individual differences has also been taken using task-free or “resting-state” studies in fMRI, in which participants monitor a fixation cross or close their eyes and endogenous variation in brain activity is measured (e.g., Fox and Raichle, 2007). In these contexts, correlation of BOLD activity is calculated across pairs of brain regions or networks of interest for a given participant, which is then correlated across participants with behavioral measures of interest. This has been done in neurologically intact participants when studying particular domains of cognition, as well as in clinical studies of particular patient groups (e.g., Finn et al., 2015; Gotts et al., 2012, 2013; Jasmin et al., 2023; Kaiser et al., 2015; Ramot et al., 2019; Rosenberg et al., 2016; Sheffield and Barch, 2016; Stevens et al., 2017; Zhu et al., 2011; for discussion, see Martin et al., 2012).

A recent study has demonstrated some practical limits on our ability to achieve this goal, at least for inter-individual differences in behavior (Marek et al., 2022, “Reproducible brain-wide association studies require thousands of individuals”; see also Elliot et al., 2020). Marek et al. (2022) examined the sample sizes needed for replication in order to associate brain measures such as cortical thickness and resting-state functional connectivity with complex behavioral measures such as overall cognitive ability and psychopathology (referred to as Brain Wide Association Studies, or BWAS). Using the largest neuroimaging datasets that are publicly available (e.g., Human Connectome Project, Van Essen et al., 2013; ABCD, Casey et al., 2018; UK Biobank, Sudlow et al., 2015) they found that effect sizes were much weaker than expected (approximately 0.1 or below), and that thousands of participants were required for robust replication. This contrasts to the typical sample size of most BWAS studies (approximately *N* = 25), explaining the lack of replicated findings. These effect sizes might be improved by restricting the samples to those of high quality (improved signal, decreased sensitivity to scanning artifacts such as motion) and restricting acquisitions to single scanning sites (e.g., Spisak et al., 2023; c.f. Tervo-Clemmens et al., 2023). Nevertheless, even if these effect sizes were doubled, the overall expectations of smaller effect sizes (an effect size of 0.2 will require approximately 200 participants to detect an effect of *p* < 0.05 at 80% power) is still that much larger samples will be required to reliably observe brain-behavior associations than those typically acquired in individual labs.

**Figure 1:**
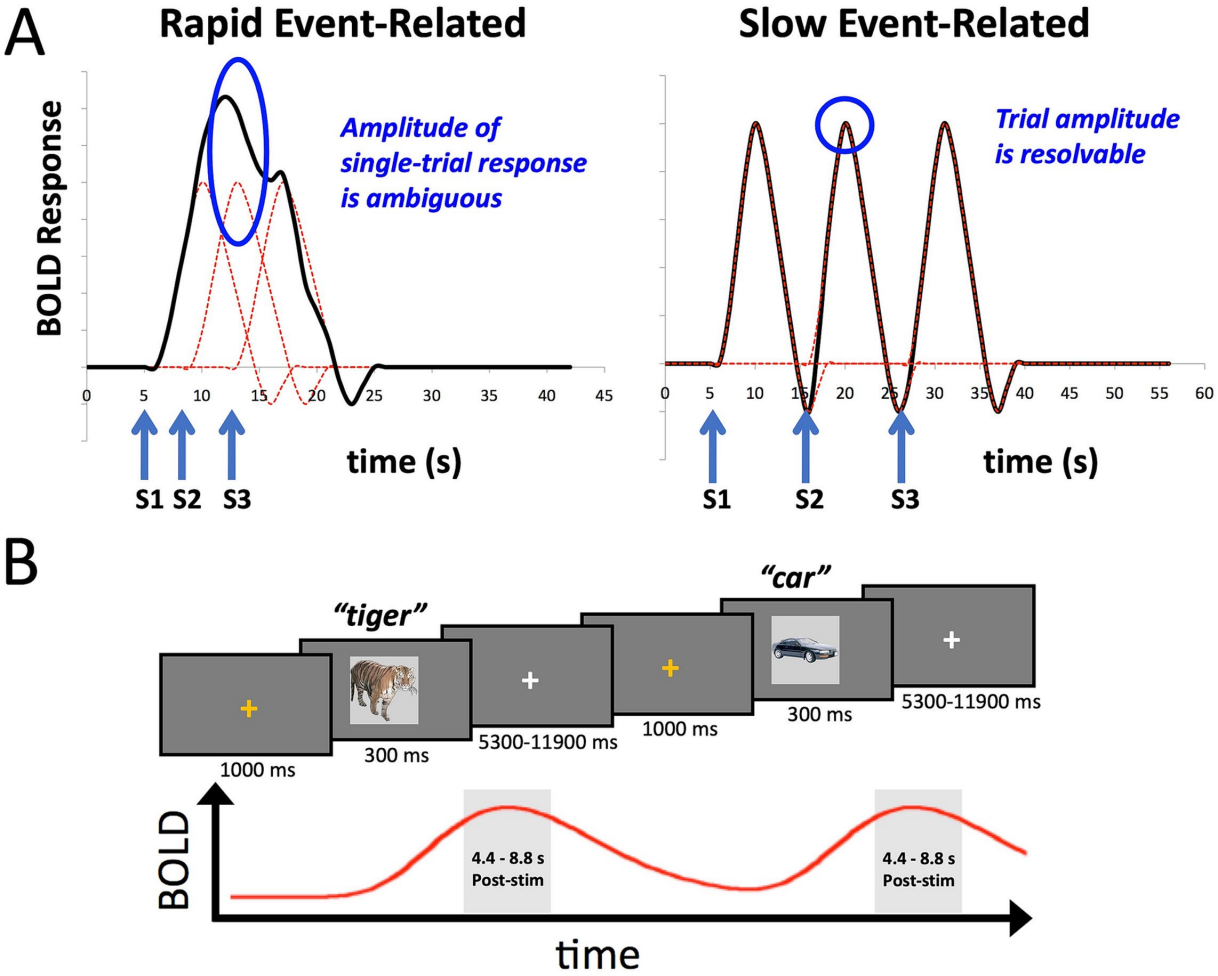
Slow event-related fMRI designs help to isolate BOLD responses on individual trials. **(A)** The plot on the left shows the idealized BOLD response to stimulus onsets S1-S3 convolved with a standard hemodynamic response function. The height of any one of the individual responses is ambiguous due to temporal overlap. The plot on the right shows that when the stimuli S1-S3 are separated by approximately 10 s each, the individual BOLD responses are better isolated, allowing estimates of the individual trial responses. **(B)** The design of the slow event-related fMRI paradigm used in the current study. An orange fixation cross was presented for 1,000 ms to alert the participant to the start of the trial. Stimuli were presented for 300 ms. Participants were instructed to overtly name each object (e.g., “tiger”), with responses recorded by an MR-compatible microphone. Stimuli were immediately followed by a white fixation cross of a variable duration (5,300–11,900 ms). Individual BOLD responses were estimated by averaging the 3rd and 4th TRs post stimulus-onset (4.4–8.8 s).

However, Marek et al. (2022) also highlighted the potential utility of certain smaller-sample neuroimaging studies for assessing brain-behavior relationships, particularly those employing within-person designs with “induced” effects (such as tasks), as opposed to resting-state functional connectivity. Such studies can have increased measurement reliability and effect sizes. Here we provide a concrete example of this alternative, utilizing a task-based fMRI design with participants overtly naming pictures of common objects. Picture naming indexes several large cognitive domains including vision, conceptual processing, language, and motor functioning (e.g., Gilmore et al., 2019; Glaser, 1992; Gotts et al., 2021; Johnson et al., 1996; Kan and Thompson-Schill, 2004). In order to estimate single-trial responses to the task, we adopt a slow-event related design rather than the more common rapid-event related design (Figure 1A; see Bandettini and Cox, 2000; Gotts et al., 2021, for discussion). This allows us to isolate better the BOLD response to individual trials for which we have a measure of behavioral performance in the task, namely response time (RT). While the temporal variation in RT is small relative to the overall time course of the BOLD signal (e.g., RT: 500–1,500 msec; BOLD signal: ~ 16 s), different trial durations are expected to manifest as different amplitudes in the BOLD response (e.g., Yarkoni et al., 2009; Rao et al., 2014; Yamasaki et al., 2017). We further utilize multi-echo imaging to aid with reducing movement artifacts from overt speech and compare this to more standard single-echo acquisition. The data presented here are taken from a previous fMRI study examining the role of task (picture naming versus recognition memory) on stimulus repetition effects (Gilmore et al., 2019). However, we have previously only reported the condition averages from this prior study—not aspects of the single-trial responses, and here, we only analyze the data from the first two scanning runs prior to any stimulus repetition (Initial Naming Phase).

Critical to the evaluation of effect sizes is some form of cross-validation to avoid inflated estimates within-sample due to overfitting (e.g., Marek et al., 2022; Spisak et al., 2023). We satisfy this requirement here in a relatively simple way, using an orthogonal effect to the effect of interest: the mean BOLD response to the task versus the correlation of trial-level BOLD responses and behavior. Selecting voxels based on the mean BOLD response across trials has no biased relationship to the estimation of the trial-to-trial variability around the mean as it covaries with an independent behavioral measure.

## Materials and methods

### Participants

Data from 40 participants (23 female) previously reported in Gilmore et al. (2019) were included in the current study. Only data from the first two scanning runs (Initial Naming Phase) of Gilmore et al. (2019) are analyzed here (out of 6 total runs), and only summary behavioral data were previously reported for these two runs (percent correct and mean reaction time, Figure 2 in Gilmore et al., 2019); all analyses presented in the current study are novel. Participants had a mean age of 24.6 years (range: 18–35), were right-handed, and were neurologically healthy native English speakers with normal or corrected-to-normal vision. Informed consent was obtained from all participants, and the experiment was approved by an NIH Institutional Review Board (protocol 93-M-0170, clinical trials number NCT00001360).

**Figure 2:**
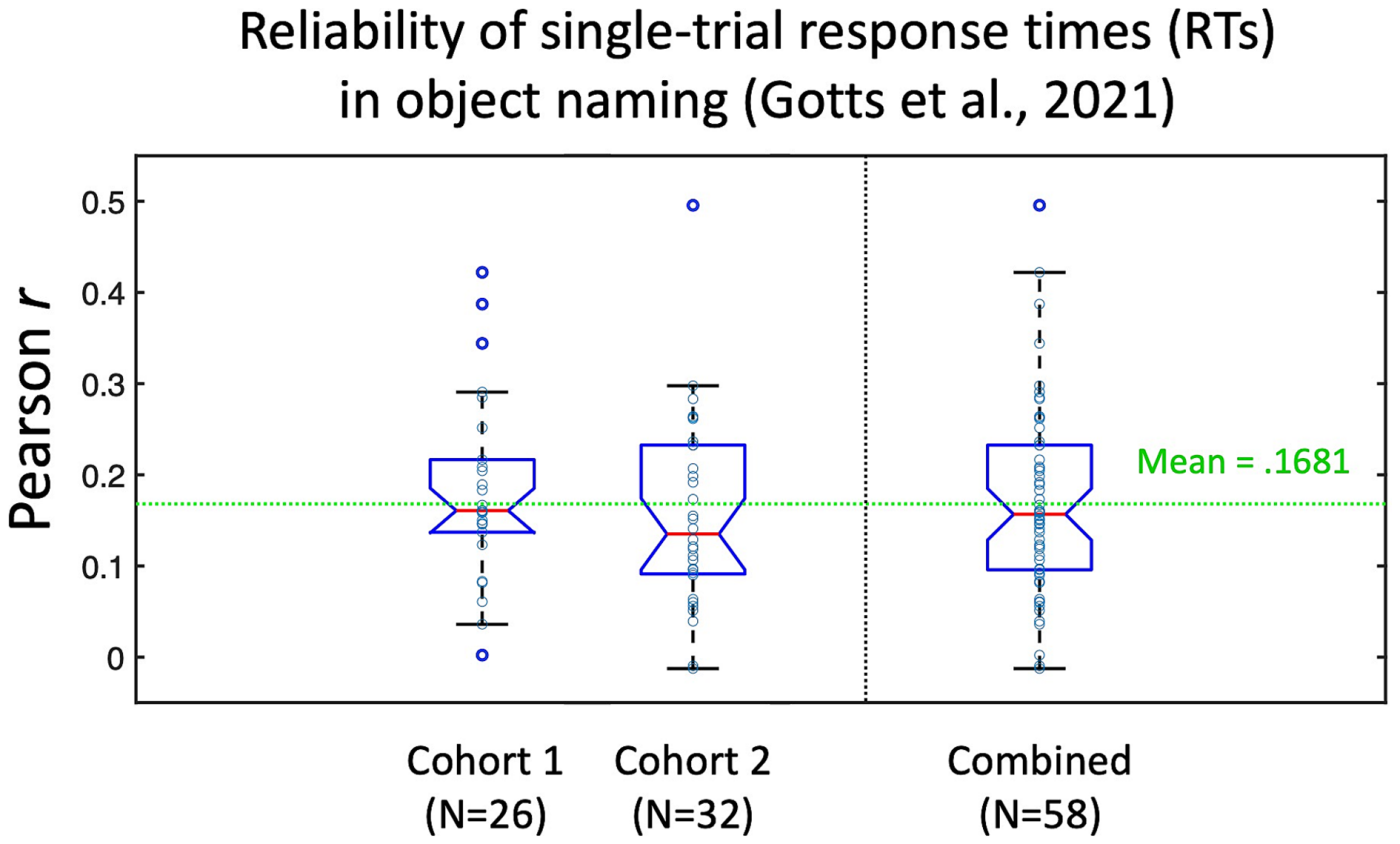
Test–retest reliability of individual picture naming trial response times (RTs) from Gotts et al. (2021). Participants in Gotts et al. (2021) named a set of 100 pictures three times. Responses to stimuli that were named correctly all three times (*N* > 80 on average per participant) were correlated across item repetitions (repetition 1 with 2, 1 with 3, and 2 with 3), with the average test–retest reliability (Pearson’s r) calculated for each individual participant. Cohort 1 corresponded to the 26/28 participants with recorded RTs in the pre-fMRI session (assigned during fMRI to the Covert Naming condition). Cohort 2 corresponded to the 32 participants who were assigned to the Overt Naming condition. Mean test–retest reliability across all 58 participants was *r* = 0.1681. Data re-plotted from Figure S1 in Gotts et al. (2021).

### Stimuli

Task stimuli consisted of 100 photographic images of common animals, plants, and man-made objects (from the Initial Naming phase of Gilmore et al., 2019, runs 1 and 2; 50 trials per run). Images were resized to 600 × 600 pixels and presented in the center of a 100 Hz MR-compatible monitor (screen resolution: 1920 × 1080 pixels) located at the head of the scanner bore and viewed through a mirror attached to the head coil. Images subtended approximately the central 8^°^ of the visual field. A fixation cross (48-point Arial type) separated image presentations, and all stimuli were presented against a gray background (RGB value of 75, 75, 75). Stimuli were presented using Presentation software (Neurobehavioral Systems) from an HP desktop computer running Windows 10.

### Task design

As discussed above in the section on Participants, data from the Initial Naming phase (runs 1 and 2) of Gilmore et al. (2019) were used in the current study. Participants overtly named images presented on the screen (Figure 1B). Each image was preceded by a 1 s orange fixation cross, which served as an onset cue for the upcoming stimulus. The image itself was presented for 300 ms and was replaced immediately by a white fixation cross for a variable period of 5,300–11,900 ms, occurring in fixed increments of the scanner repetition time (TR = 2,200 s). Participants were instructed to name aloud each image as quickly and accurately as possible. Responses were spoken into an MR-compatible microphone that was attached to the head coil and was placed 3–5 cm from the participant’s mouth.

### Audio recording equipment

Participants spoke all responses into an Octo-Acoustics FOMRI-III NC MR-compatible microphone with built-in noise cancelation. Audio signals from this microphone were routed into an M-Audio FastTrack Ultra 8-R USB audio interface, which in turn was connected to a Dell Precision M4400 laptop. Responses were recorded as *.wav* files using Adobe Audition. In addition to a spoken audio recording, the stimulus presentation computer sent out a square wave pulse at the onset of each picture presentation that was captured on a parallel audio track by the recording laptop. A Matlab program (written by SJG) calculated the time difference between the square wave pulse onset and the voice response onset in each trial, allowing for the calculation of voice onset reaction times (RTs).

### MRI data acquisition

All images were acquired with a General Electric Discovery MR750 3.0 Tesla scanner, using a 32-channel head coil. A high-resolution T1 structural image was obtained for each participant (TE = 3.47 ms, TR = 2.53 s, TI = 900 ms, flip angle = 7^°^, 172 slices with 1 mm^3^ isotropic voxels). Functional images were acquired using a BOLD-contrast sensitive multi-echo echo-planar sequence [Array Spatial Sensitivity Encoding Technique (ASSET) acceleration factor = 2, TEs = 12.5, 27.7, and 42.9 ms, TR = 2,200 ms, flip angle = 75^°^, 64 × 64 matrix, in-plane resolution = 3.2 mm × 3.2 mm]. Whole-brain EPI volumes of 33 interleaved, 3.5 mm-thick oblique slices (manually aligned to the AC-PC axis) were obtained every 2,200 ms.

### fMRI data preprocessing

fMRI data were processed using AFNI (Cox, 1996) to reduce noise and facilitate across-participant registration. Initial preprocessing steps included: (1) eliminating the first four TRs of each run to allow for steady-state magnetization (3dTcat), (2) despiking of time series in each voxel (3dDespike) by squashing outlying time points to within 4 standard deviations of the mean, (3) adjusting for slice-time acquisition (3dTshift), and (4) volume registration of each TR to the initial kept frame from the first run. After these preliminary steps, data from all three acquired echoes were used to remove additional noise sources with multi-echo independent component analysis (ME-ICA, Kundu et al., 2012, 2013; implemented as meica.py within AFNI). In brief, this procedure initially uses a weighted averaging of the different echo times to reduce thermal noise. Subsequently, spatial ICA and the known linear properties of T2* signal decay are used to separate putative BOLD from non-BOLD components (including those having to do with head motion, hardware artifacts, etc.). Components were identified and classified automatically using the default options present in AFNI’s meica.py and tedana.py. Optimally combined (OC) data, multi-echo data without additional ME-ICA denoising, were generated by taking a weighted summation of the three echoes using the exponential weighting approach for T2* in Posse et al. (1999). Single-echo estimates simply utilized the middle echo datasets (at TE = 27.7 ms), with this TE chosen *a priori* to conveniently approximate the TE needed to optimize T2* contrast. All three preprocessing versions (ME-ICA, OC, and single-echo) were converted to units of percentage signal change (dividing the voxelwise timeseries by their corresponding means) and were then aligned to the skull-stripped anatomical image (integrated as part of meica.py for multi-echo data and using align_anat_epi.py for the single-echo data), resampled to 3 mm^3^ isotropic voxels and linearly transformed into Talairach and Tournoux (1988) atlas space. No additional spatial smoothing was applied to any of the three pipelines.

### fMRI data analyses

#### GLM analyses

Functional scans for each run consisted of 237 TRs and 50 stimuli, which after discarding the initial 4 TRs amounted to 233 TRs (8 min, 32.6 s per run). As discussed above, only the 1st two runs (Initial Naming) from Gilmore et al. (2019) are analyzed in the current study. All runs had transient motion (AFNI’s @1dDiffMag) <0.3 mm/TR. Traditional task analysis was performed on all 3 preprocessing conditions (ME-ICA, OC, and single-echo) using a General Linear Model (GLM) (AFNI’s 3dDeconvolve), in which the data at each timepoint are treated as the sum of all effects thought to be present at that timepoint, plus an error term. The GLM included a 4th order polynomial baseline, one stimulus condition for correct trials and one condition for error trials, both modeled with TR-specific TENT regressors (over 6 time points: 0, 2.2, 4.4, 6.6., 8.8, and 11.0 s) to empirically estimate the BOLD response in each voxel across the two runs. This approach assumes that all stimuli in the single condition share one response shape, although it does not presume the shape of that response. For purposes of statistical testing (both within and across participants), response magnitudes to correct trials were estimated by averaging the 3rd and 4th time points of the TENT regressors (beta coefficients) in each voxel, corresponding to the expected peak of the BOLD signal at 4.4–8.8 s post-stimulus onset. Group-level effects of stimulus condition (Stimulus versus a baseline of 0 during fixation) were assessed with one-sample *t*-tests in each voxel, with multiple comparisons corrected by False Discovery Rate to *q* < 0.05 (Genovese et al., 2002). For replication tests, stimulus effects for participants were randomly divided into two halves (*N* = 20 participants in each), with tests conducted separately in each half and corrected for multiple comparisons to FDR *q* < 0.05 prior to conjunction (see Nichols et al., 2005, for discussion). This process was then repeated 100 times, with the likelihood of replication across the halves calculated.

#### Correlation of single-trial BOLD amplitudes with response time

Single-trial estimates of the BOLD response were not explicitly modeled. Rather, the assumed peak response (average of the 3rd and 4th TRs post-stimulus) to each stimulus was notched out of the overall time series (AFNI’s 3dTcat) in each voxel after first detrending the time series in each run with a 4th order polynomial (using AFNI’s 3dDetrend). Pearson correlations between the trial-wise BOLD responses and the trial-wise response times on correct trials were then calculated in each voxel (up to 100 correct trials per participant) and Fisher *z’*-transformed [*atanh(r)*] to yield normally distributed values. Statistical testing could then be performed in each voxel at the single-participant level using the number of trials (traditional *r*-test) or at the group level by conducting one-sample *t*-tests on the mean trial-level correlation across participants, with multiple comparisons corrected by False Discovery Rate to *q* < 0.05 (see Yamasaki et al., 2017, for a similar approach in the Stop Signal Reaction Time task). For replication tests, correlation tests for participants were randomly divided into two halves (*N* = 20 participants in each), with tests conducted separately in each half and corrected for multiple comparisons to FDR *q* < 0.05 prior to conjunction (see Nichols et al., 2005, for discussion). This process was then repeated 100 times, with the likelihood of replication across the halves calculated.

In addition to whole-brain voxelwise tests, group-level effects of BOLD-RT correlations were also assessed by selecting on an orthogonal effect, namely the GLM task response. The two effects are orthogonal because selecting on a value of the mean stimulus response does not bias the correspondence between individual trials varying around the mean and the independently acquired trial-level behavioral measure (i.e., RT). Effects of BOLD-RT correlation were assessed at the group level in 4 conditions: a whole-brain mask, the top 10,000 voxels in the task-positive stimulus response, the top 5,000 voxels, and the top 1,000 voxels. The selective masks were constrained to be task-positive to avoid the potential for positive–negative cancelation of the BOLD-RT correlation when estimating the magnitudes. For each mask, BOLD-RT correlations were averaged across the voxel set for each participant. Statistical tests were carried out using a linear mixed effects (LME) model with within-participant factors of Preprocessing (ME-ICA, Optimally Combined, Single-Echo) and Voxel Mask (top 1,000, top 5,000, and top 10,000 voxels in the mean task response) and Participant treated as the random intercept. Post-hoc paired comparisons were conducted with paired *t*-tests across participants, and multiple-comparisons were corrected by FDR to *q* < 0.05.

#### Effect size and sample size estimations

Effect sizes for one-sample *t*-tests were estimated using Cohen’s *d*, which is simply the mean divided by the standard deviation of the tested population of values. Effect sizes for Pearson correlation coefficients here are simply the Fisher *z’*-transformed Pearson *r*-values. Given these specifications, we used the formula given by Lachin (1981; Eq. 8) to estimate the needed sample size, *N*, to detect effects at *p* < 0.05 and 80% power (a Type-II error rate of 0.2):

$$N={\left(\frac{Z_{\alpha }+Z_{\beta }}{d}\right)}^{2}$$

where *Zα* is the value of the two-tailed normal distribution corresponding to *α* = 0.05, *Z_β_* is the one-tailed normal distribution corresponding to the power level (0.8), and *d* is the effect size. For fixed *p* < 0.05 and 80% power, this equation simplifies to:

$$N=\frac{7.849}{d^{2}}$$

These analyses were carried out on BOLD-RT correlations when selecting voxels on the orthogonal effect of the mean task response in order to avoid inflated within-sample biasing (for discussion, see Spisak et al., 2023; Tervo-Clemmens et al., 2023). Sampling distributions for the effect sizes were estimated from the measured data through bootstrap resampling (10,000 iterations), which permitted calculation of 95% confidence limits (2.5%-ile and 97.5%-ile of the bootstrapped distributions).

#### Dependence on number of trials

The impact of trial number on the ability to observe BOLD-RT correlations was investigated by including the first *X* trials from each participant (5, 10, 20, 30, 40, 50, or all correct trials—mean = 91.59 trials across participants, range = 78–99 trials) and recalculating the BOLD-RT correlations, effect sizes and needed sample sizes. Comparisons of mean BOLD-RT correlations across the 7 trial number conditions were carried out by paired t-tests across participants, with multiple comparisons corrected by FDR to *q* < 0.05. Comparisons of Cohen’s *d* effect sizes across the 7 trial number conditions were carried out through bootstrap resampling (10,000 iterations). On a given iteration, participants were randomly selected with replacement to equal 40 participants. Effect sizes were then calculated for each trial condition and the difference in effect sizes for each pair of conditions was recorded. Over 10,000 iterations, the *p*-value for a given comparison corresponded to the percentile rank of 0 in the distribution, converted to 2-tailed *p*-values by multiplying the 1-tailed *p* by 2.0. Multiple comparisons were corrected by FDR to *q* < 0.05.

## Results

In the current experiment, we evaluate the feasibility of detecting brain-behavior correlations at a trial-level per participant during object naming. Since test–retest reliability of a measure constrains its possible correlation with other measures (e.g., Nunnally, 1959), it is useful to estimate these values when possible. While we do not have ready estimates of test–retest reliability of the single-trial fMRI BOLD responses from prior studies, the pre-fMRI behavior-only phase of Gotts et al. (2021) involved each participant naming a set of 100 pictures three times (using the same pictures as in the current study, but with different participants). From this experiment, we are able to estimate the test–retest reliability of our response time (RT) measure by correlating the single-trial RTs across different naming attempts of the same pictures by each person. It is important to note that the very act of repeating a stimulus will alter behavioral and neural responses to it, leading to faster RTs and decreases/increases in BOLD (see Gilmore et al., 2019, for discussion). The decreased range of RTs to repeated stimuli in the presence of measurement noise/variability may therefore lead to slightly decreased estimates of test–retest reliability, but these empirical benchmarks still provide useful context. The test–retest estimates are shown in Figure 2 for the 58 participants from Gotts et al. (2021) with pre-fMRI naming data (Cohort 1 participants were assigned to the Covert Naming condition during fMRI for this experiment, Cohort 2 participants were assigned to the Overt Naming condition; all participants performed Overt Naming in the pre-fMRI session). Overall, participants had very low test–retest reliability of the single-trial RTs (mean = 0.1681), although values covered a large range for individual participants (−0.012–0.496). This suggests that many trials would be required for BOLD-RT correlations to be significant for the average participant (for an effect size of 0.1681, the expected *N* in trials to detect an effect at *p* < 0.05 with 80% power is 278 trials; Lachin, 1981), and many more still would be required if the test–retest reliability of the single-trial BOLD responses is low and/or if the true BOLD-RT correlation is low. Based on this prior experiment, we should not expect the mean BOLD-RT correlation across participants observed in the current study to be much larger than 0.17.

**Figure 3:**
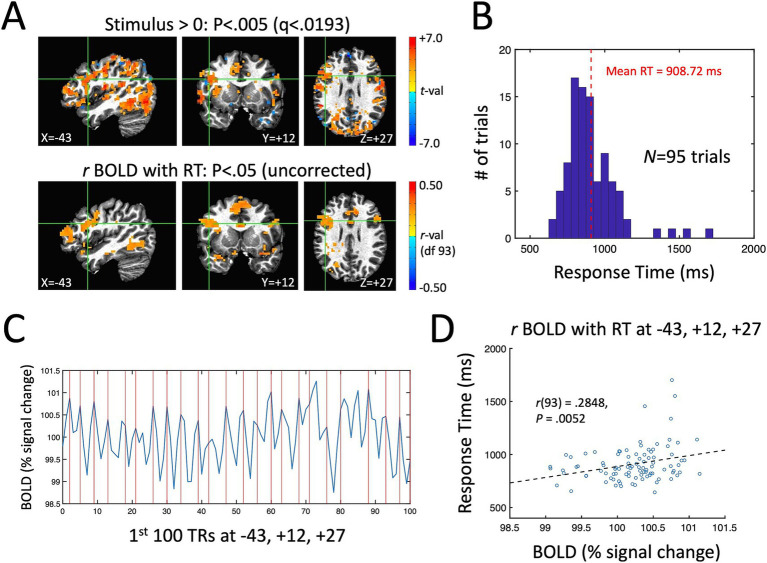
Single-participant example of the measured task effect and BOLD-RT correlation effect during picture naming. **(A)** Top row shows locations where the BOLD response was significantly above (below) zero in red (blue) colors *p* < 0.005 (corrected by FDR to *q* < 0.0193). Bottom row shows locations where the correlation between BOLD and RT across 95 correct trials is greater than (red colors) or <0 (blue colors) (*p* < 0.05, uncorrected). As in prior studies of picture naming, prominent task-related activity is seen in occipito-temporal and frontal cortex. **(B)** Distribution of RTs across trials for this participant, with mean RT of 908.72 ms. **(C)** The first 100 TRs from a voxel in left frontal cortex highlighted by the green crosshairs in **(A)** (T–T coordinate −43, +12, +27). The x-axis shows TR number and the y-axis shows the BOLD responses in units of % signal change. The expected onsets of the BOLD peaks on individual trials are shown with vertical red lines at 4.4 s post stimulus-onset. On average, there is good correspondence visually between the expected and actual BOLD peaks on single trials. **(D)** The correlation of BOLD (% signal change) and RT (ms) on individual trials is shown for the highlighted voxel in (A) and (C). The scatterplot of individual trials reveals a positive correlation, with slower RTs associated with higher amplitude BOLD responses [*r*(93) = 0.2848, *p* = 0.0052].

A single-participant example of the main quantities of interest in the current study for the ME-ICA processed data is shown in Figure 3. Figure 3A shows the stimulus effect (Stimulus vs. a baseline of 0 during fixation) on the BOLD response in the top panel (*p* < 0.005, FDR-corrected to *q* < 0.0193) and the correlation between BOLD and RT across trials in the bottom panel (*p* < 0.05, uncorrected). Despite the expected low correlations between BOLD and RT, the uncorrected map is quite similar to the overall task response—and similar to the brain regions known to be involved in picture naming from prior studies (e.g., left lateral frontal cortex, the fusiform gyrus bilaterally and the anterior cingulate; Gotts et al., 2021). As in prior picture naming studies, the distribution of response times for this participant ranges from 600 ms up to 1,500 ms with a mean of approximately 909 ms (Figure 3B). In Figure 3C, we have shown the first 100 TRs of the BOLD response (220 s) from a voxel in left frontal cortex showing both a significant task response and a correlation between BOLD and RT (highlighted by the green crosshairs in Figure 3A). Vertical red lines are placed at the beginning of the expected BOLD peak (4.4 s after stimulus onset) for each correctly named stimulus during that period (*N* = 23 correct responses). Visually, there is quite good correspondence between the BOLD peaks and the expected onsets for this voxel. Finally, a scatterplot of the single-trial BOLD peaks (x-axis) with the single-trial RTs (y-axis) is shown in Figure 3D for the same highlighted voxel (at T–T coordinate −43, +12, +27). As in previous studies examining BOLD-RT correlations in task-positive regions (e.g., Rao et al., 2014), there is a positive slope between BOLD and RT, with slower trials having larger BOLD responses [*r*(93) = 0.2848, *p* = 0.0052].

### Group effects of task and BOLD-RT correlations

**Figure 4:**
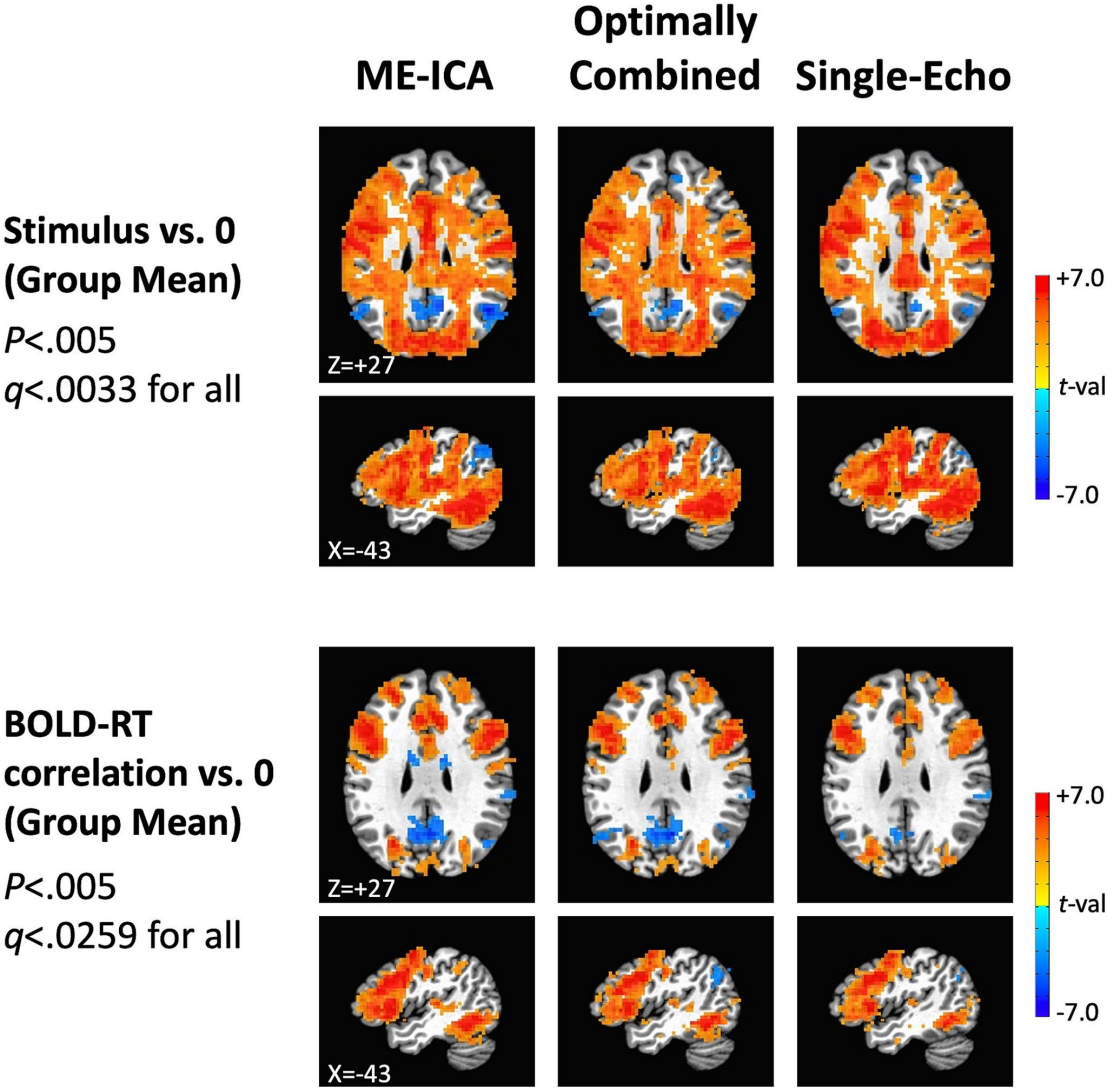
Group-level effects of task response and BOLD-RT correlations by trial. In the top rows, the locations where the mean BOLD response to picture naming trials differed from zero across participants in the three preprocessing conditions (ME-ICA, Optimally Combined, and Single-Echo) are shown, with above-baseline responses shown in red and below-baseline (task-negative) responses shown in blue (*p* < 0.005, *q* < 0.0033 for all). Mean task-responses were quite similar across all three preprocessing conditions. In the bottom rows, the locations where the group mean across the participant-level BOLD-RT correlations (calculated by trial) differs from zero (positive correlations in red, negative correlations in blue) are shown by preprocessing condition (*p* < 0.005, *q* < 0.0259 for all). The correlations were more spatially extensive and higher amplitude in the ME-ICA condition than in the Optimally Combined for Single-Echo conditions.

Next, we turn to the effects of picture naming on the mean BOLD response at the group level for the three preprocessing conditions (ME-ICA, Optimally Combined, and Single-Echo), as well as the group average of the trial-level correlations between BOLD and RT. The top panels of Figure 4 show voxels where the group mean task response is significantly different from zero (either above-baseline in red or below baseline in blue) (*p* < 0.005, *q* < 0.0033 across all three conditions). The task positive (above-baseline) responses here accord well with those previously described in Gotts et al. (2021), with positive BOLD responses throughout visual, temporal, somatomotor and prefrontal cortex. There are also significant task-negative responses in all three preprocessing conditions in regions of the canonical “default mode” network (e.g., Fox et al., 2005). Tests of the group mean of the trial-level BOLD-RT correlations versus zero are shown for the three preprocessing conditions in the bottom panels of Figure 4. Despite the expected weak within-participant BOLD-RT correlation values (based on the results in Figure 2), the corresponding group-level tests of the mean of these values across participants yield robust effects, with positive mean BOLD-RT correlations in frontal, temporal, and parietal regions and negative mean BOLD-RT correlations in regions of the default mode network (*p* < 0.005, *q* < 0.0259 across all three conditions).

### Effect of preprocessing and mean task response on trial-level BOLD-RT correlations, effect sizes, and needed sample sizes

In order to avoid inflated estimates of trial-level BOLD-RT correlations and their corresponding effect sizes, we selected voxels based on an orthogonal effect, namely the mean task response across participants. Selecting on the mean value of the BOLD response pools responses across all trials and does not differentially bias particular trials, nor does it bias the correspondence of individual trial values to independent behavioral measures (RT). However, if both measures index the same cognitive ability (picture identification), they should nevertheless identify similar sets of voxels. In other words, voxels with a larger mean BOLD response might be expected to be similar to those with a large BOLD-RT correlation if the underlying neural activity is engaged in picture identification. We therefore thresholded the mean BOLD response across participants at several different levels: the top 10,000 voxels with a task-positive response (voxels with the largest group-level effect size), the top 5,000 voxels, and the top 1,000 voxels. We also included a whole-brain mask for comparison. Note that task-positive voxels should tend to have BOLD-RT correlations with the same slope (positive; see Figure 4) so that positive/negative effects do not cancel in the estimates (whereas this is possible in the whole-brain mask).

**Figure 5:**
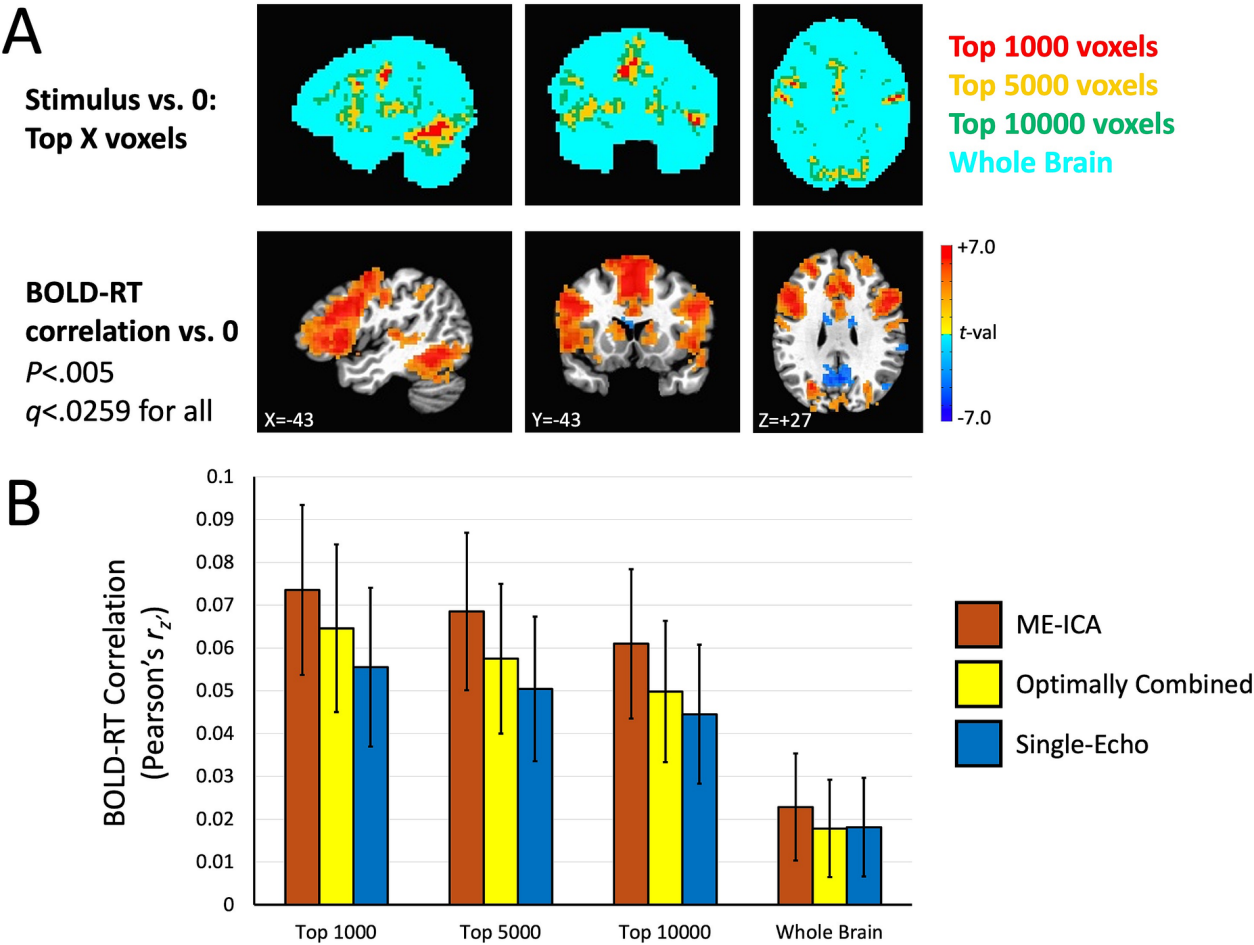
BOLD-RT correlation magnitudes when selected on the orthogonal effect of mean task activation. **(A)** Voxel masks used to estimate BOLD-RT correlations are shown in the top row. The largest responses in the group mean BOLD response were selected at differing levels: the top 1,000 voxels (red), the top 5,000 voxels (yellow), and the top 10,000 voxels (green), with a whole-brain mask (cyan) for comparison. The voxel masks are overlapped such that the top 1,000 voxels are also part of the top 5,000 voxel mask and the top 10,000 voxels masks (and the top 5,000 voxel mask is part of the top 10,000 voxel mask). The bottom row shows where the group mean BOLD-RT correlations differ from zero (shown also in Figure 4). **(B)** The voxel masks in **(A)** were applied to the individual participants’ BOLD-RT correlation maps. The BOLD-RT correlations (Fisher’s *z’*-transformed Pearson *r*-values, *r_z’_*) were averaged across all voxels in a given mask, with the mean across participants compared to zero (and among the conditions). BOLD-RT correlations were larger for voxels with larger amplitude task responses (top 1,000 versus top 5,000 or top 10,000 voxels) and larger for multi-echo acquisition than for single-echo (ME-ICA and Optimally combined larger than Single-Echo), with ME-ICA BOLD-RT correlations also greater than Optimally Combined within the multi-echo conditions. Error bars represent the 95% confidence limits of the group means.

The results are shown in Figure 5. Figure 5A shows the masks with the different thresholds in the top panel along with the group mean effect of the BOLD-RT correlations (using the ME-ICA processed data as an example). The top voxels in the task response do appear to correspond to the larger BOLD-RT correlations spatially. Figure 5B shows the BOLD-RT correlations, averaged within the respective masks and across participants for the three different preprocessing conditions (error bars depict the 95% confidence interval of the mean). The first thing to note is that the individual BOLD-RT correlation values are indeed capped (as expected) by the test–retest reliability estimates of single-trial RTs shown in Figure 2 (*r* = 0.17). All of the individual conditions (including those for the whole-brain mask) are nevertheless significantly different from zero when considering the group means of the conditions (*p* < 0.0029, *q* < 0.05 for all). The three Preprocessing conditions (ME-ICA, Optimally Combined, Single-Echo) and the selective Voxel Mask conditions (top 1,000, 5,000, and 10,000 voxel masks) were entered as factors in a linear mixed effects (LME) model with Participant as the random intercept. Significant main effects of Preprocessing [*F*(2,312) = 46.73, *p* = 1.77×10^−18^, *q* < 0.05] and Voxel Mask [*F*(2,312) = 37.67, *p* = 2.22×10^−15^, *q* < 0.05] were observed, with no significant interaction between the factors [*F*(4,312) = 0.16, *p* > 0.9]. Underlying the main effect of Preprocessing, ME-ICA had larger BOLD-RT correlations than both Optimally Combined [paired *t*(39) = 3.525, *p* < 0.0011, *q* < 0.05] and Single-Echo conditions [paired *t*(39) = 4.080, *p* < 0.0003, *q* < 0.05], and Optimally Combined had greater BOLD-RT correlations than Single-Echo [paired *t*(39) = 2.189, *p* < 0.0347, *q* < 0.05]. Underlying the main effect of Voxel Mask, the top 1,000 task voxels yielded higher BOLD-RT correlations than both the top 5,000 [paired *t*(39) = 3.128, *p* < 0.0034, *q* < 0.05] and top 10,000 masks [paired *t*(39) = 5.096, *p* < 1.0×10^−5^, *q* < 0.05], and the top 5,000 task voxels yielded higher BOLD-RT correlations than the top 10,000 mask [paired *t*(39) = 8.785, *p* < 1.0×10^−10^, *q* < 0.05]. Thus, ME-ICA denoising improved BOLD-RT correlations relative to multi-echo acquisition without additional ICA denoising (or to single-echo), and there was also an advantage to multi-echo acquisition over single-echo acquisition (when neither has additional denoising applied). Similarly, thresholding higher on the mean task response led to higher BOLD-RT correlations, with the highest BOLD-RT correlations observed when using the top 1,000 voxels of the task response.

**Figure 6:**
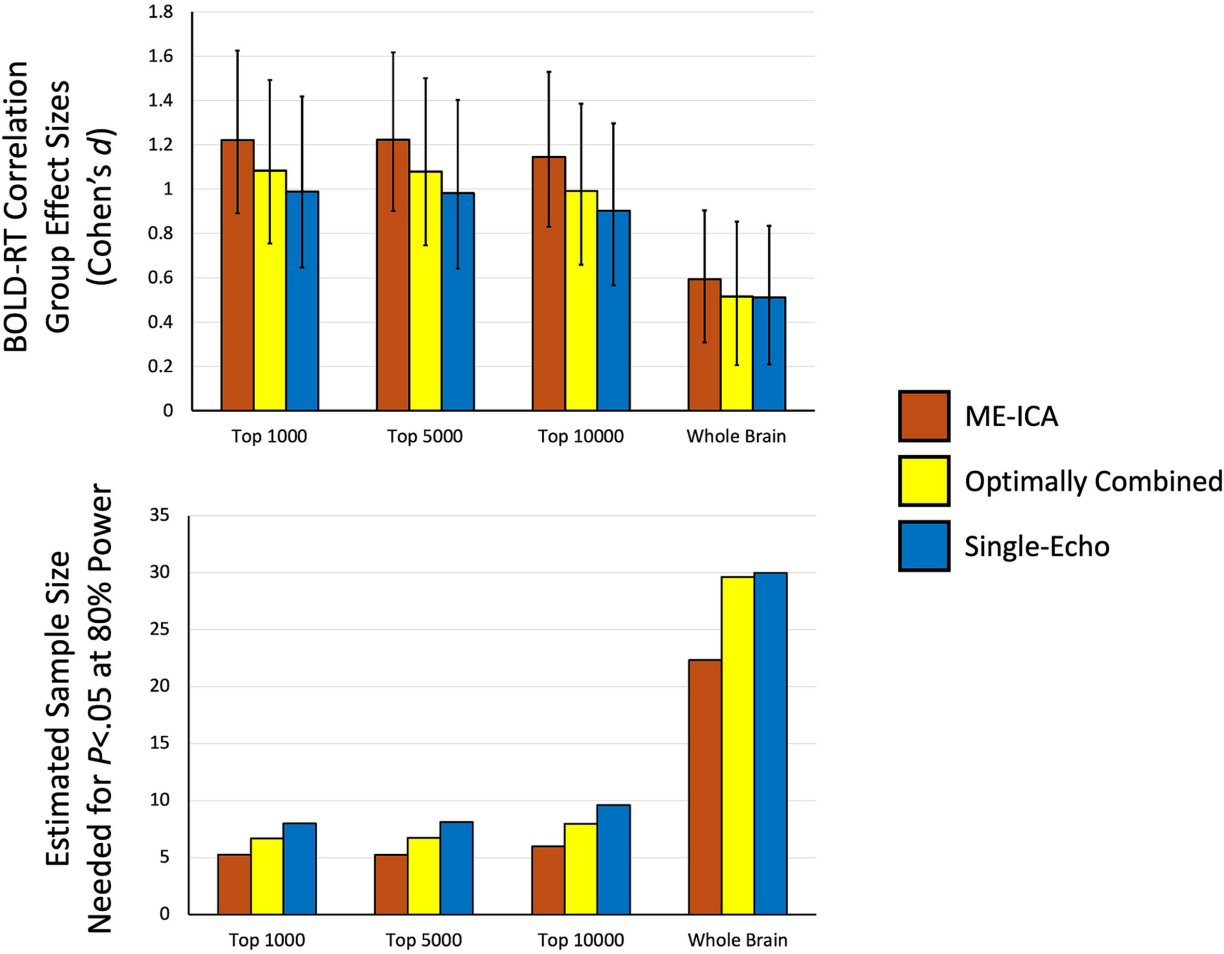
Effect sizes and estimated sample sizes needed to detect group-mean BOLD-RT correlations. The top panel shows the effect sizes (Cohen’s *d*) of the group-level effects that correspond to the mean of single-participant values shown in Figure 5B. Bootstrap resampling (10,000 iterations) permitted estimates of the confidence limits on the group-level effect sizes (errors bars represent the 95% confidence limits of the bootstrapped samples). While the individual participant BOLD-RT correlation effect sizes (by trials) are <0.1 for all conditions, the effect sizes of the corresponding group-mean effects are approximately 1.0. The bottom panel shows the estimates of the sample sizes needed to detect the group-level effects (from top panel) at *p* < 0.05 with 80% power. All of the selective voxel conditions are estimated to require 10 participants or fewer to find effects.

The effect sizes of the BOLD-RT correlations shown in Figure 5B are small when considering the individual values (a mean correlation of approximately 0.0734 in the top 1,000 voxels condition of the ME-ICA preprocessing). However, when using these values as data themselves in a group-level analysis, the mean across participants is robustly different from zero. Therefore, we next characterized the effect sizes of the group-level effects. Each condition from Figure 5B is re-plotted in the top panel of Figure 6 as group-level effect sizes (the mean divided by the standard deviation of the single-participant values), with error bars representing the 95% confidence intervals obtained with bootstrap resampling (10,000 iterations). While the individual values of BOLD-RT correlation are quite weak (below 0.1), they are reliably above zero across participants, and their corresponding group-level effect sizes for the selective voxel masks have Cohen’s *d*’s of approximately 1.0 (the highest being the top 1,000 voxel mask for ME-ICA preprocessing: mean *d* = 1.221; the lowest being the top 10,000 voxel mask for Single-Echo preprocessing: mean *d* = 0.9031). These higher effect sizes have a large impact on the sample sizes needed to detect an effect at *p* < 0.05 with 80% power (shown in the bottom panel of Figure 6). Using the formula for sample size calculation given by Lachin (1981) (see Materials and Methods), the needed sample size is fewer than 10 participants for all of the selective voxel masks and all three preprocessing conditions. If one instead considers a slightly more rigorous threshold of *p* < 0.05 with 90% power, the needed sample size only increases to 13 participants for these conditions.

**Figure 7:**
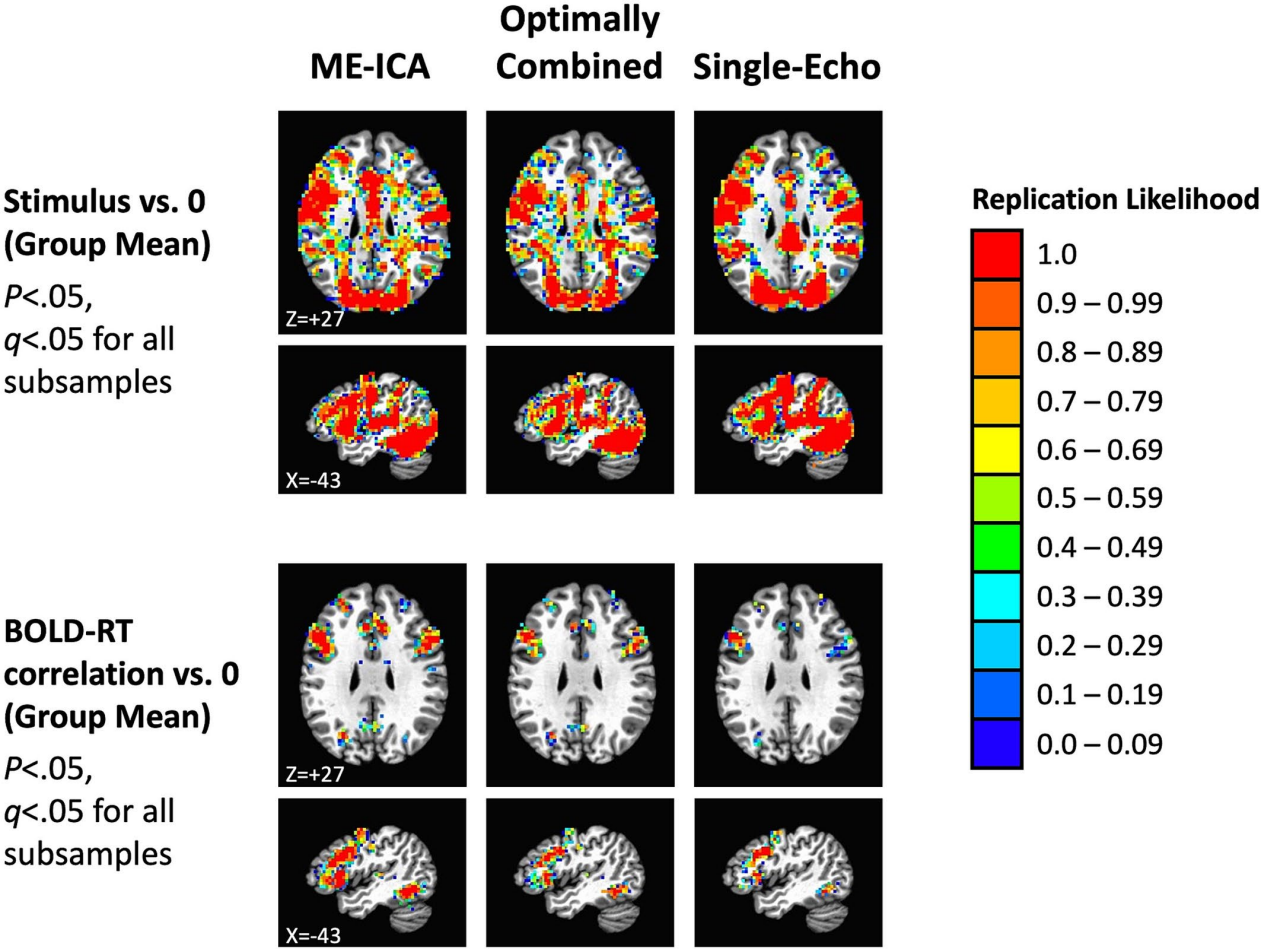
Replication of mean task effects and group-mean BOLD-RT correlation effects for the three preprocessing conditions across random independent subsamples of participants (the total sample of 40 participants randomly divided into 2 subsamples of 20 participants each, repeated 100 times). The top rows show the locations where the mean BOLD response during picture naming replicates across subsamples of participants (*p* < 0.05, *q* < 0.05 for each subsample). The color scale indicates the average likelihood of replication across the 100 random iterations, with red voxels marking locations with a replication likelihood of 1.0. The spatial extent of replication was similar across the three preprocessing conditions for the mean BOLD response to task. The bottom rows show the locations where the group mean of the participant-level BOLD-RT correlations differs from zero and replicates across subsamples (*p* < 0.05, *q* < 0.05 for each subsample). There was a greater spatial extent of replication for ME-ICA preprocessing relative to the other two conditions.

These results suggest that our total sample size (*N* = 40) should be larger than needed to observe replications across independent subsamples of the data. We investigated this by dividing participants into two equal groups (*N* = 20 in each) by random assignment, conducting the tests separately in each subsample, correcting for multiple comparisons (for all *p* < 0.05, FDR *q* < 0.05), and then forming conjunctions of the results to detect replications across the subsamples. This process was then repeated over 100 total iterations to estimate the overall likelihood of replication in each voxel (ranging from 0.0 to 1.0). Replication likelihood across random subsamples is shown in Figure 7 for the three preprocessing conditions (effects of mean task response in the top panel and mean BOLD-RT correlation in the bottom panel). As anticipated from the results shown in Figure 6, all effects do indeed replicate, with similar extents observed for the mean effect of task across preprocessing conditions (red voxels indicate which voxels show replication across all 100 random iterations). More spatially extensive replication was observed for the mean effect of BOLD-RT correlations when using ME-ICA preprocessing than for the other two preprocessing conditions.

### Effect of number of trials included on trial-level BOLD-RT correlations, effect sizes and needed sample sizes

**Figure 8:**
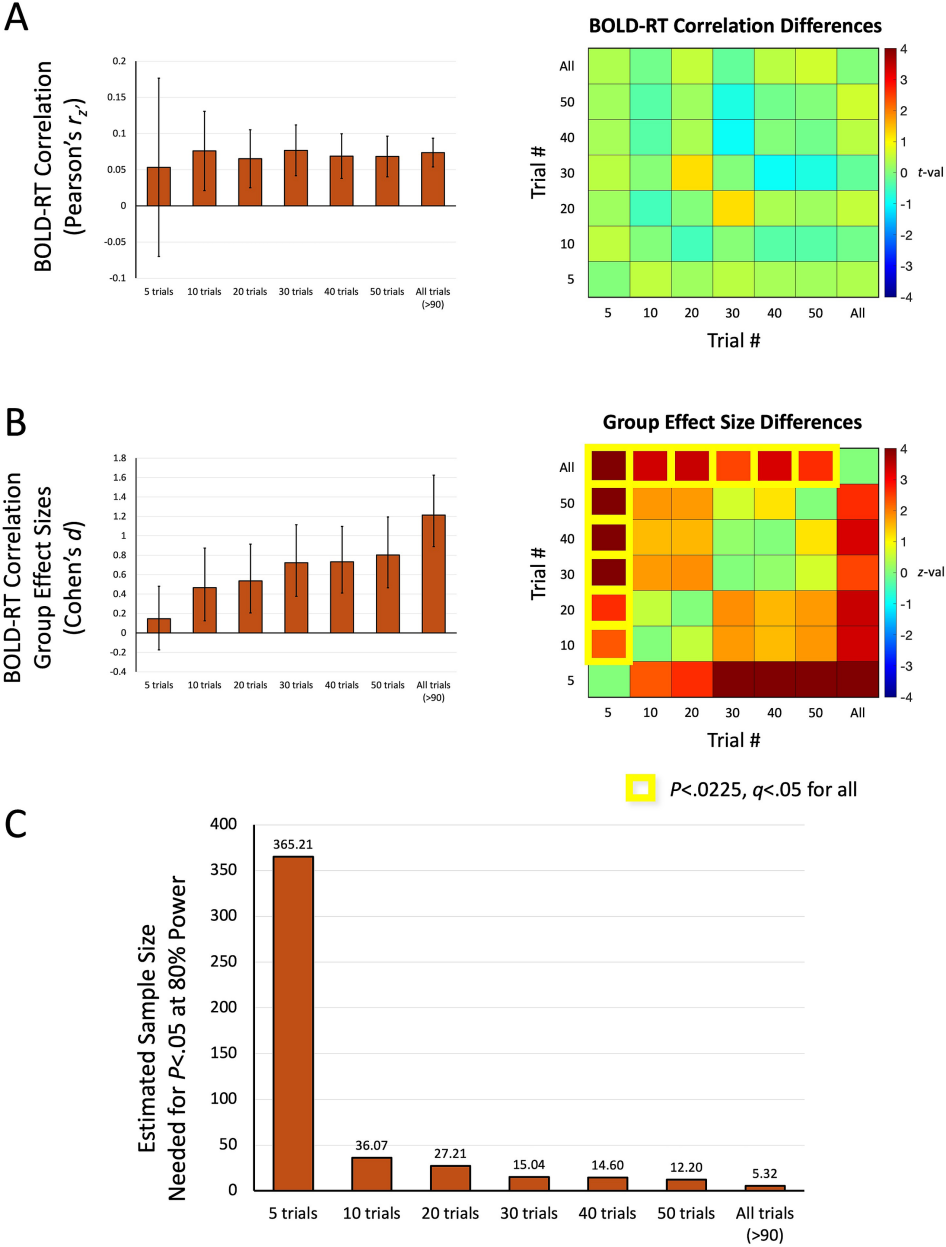
The effect of number of trials on BOLD-RT correlations, effect sizes, and needed sample sizes. **(A)** The left panel shows the mean of the participant-level BOLD-RT correlations for the ME-ICA preprocessing condition (top 1,000 voxel mask) with different numbers of trials included per participant (ranging from 5 trials to All trials, on average > 90 trials per participant). Error bars reflect the 95% confidence limits on the means. The right panel shows that the mean BOLD-RT correlation value across participants does not differ significantly with different numbers of trials included (*t*-values reflect one-sample t-tests on the difference of the condition with the larger number of trials minus the smaller number of trials; all values non-significant). Rather, the variability around the mean shrinks with more trials included. **(B)** Due to the shrinking variability of the individual participant-level values around the mean with larger numbers of trials included, the effect sizes of the corresponding group-level effects increase with a greater number of trials. In the left panel, the error bars reflect the 95% confidence intervals of the bootstrapped samples (10,000 iterations). The right panel shows that the trial conditions do differ significantly in the effect size estimates, with 5 trials yielding effect sizes smaller than all other conditions and All trials yielding effect sizes larger than all other conditions (*p* < 0.0225, *q* < 0.05 for all; yellow squares denote trial condition combinations that differ and that survive FDR-correction over all comparisons). The *p*-values of the condition differences estimated with bootstrap resampling were converted to *z*-values (shown in the matrix), indicating the *z*-test of the condition with the larger number of trials minus the condition with the smaller number of trials. **(C)** The mean effect sizes from (B) were used to estimate the needed sample sizes to detect an effect to *p* < 0.05 at 80% power. For 5 trials, more than 360 participants would be needed to detect an effect, whereas this drops to fewer than 40 participants for 10 trials, fewer than 30 participants for 20 trials, and down to fewer than 10 participants when using All trials (>90 per participant).

The results discussed above highlight a counterintuitive situation. A statistical test conducted for each participant over trials should fail to be significant (much less corrected for multiple comparisons) due to small effect sizes (<0.1). However, when utilized as a datum rather than a statistical test, these quantities are reliably different from zero at the group-level. What this suggests is that while the values of the BOLD-RT correlations for each participant over trials are not strong, they are approaching stable values with the numbers of trials included in this experiment (on average 91.59 correct trials per participant). We investigated this issue further by analyzing different numbers of trials per participant (5, 10, 20, 30, 40, 50, and all trials) and recalculating the BOLD-RT correlations, group effect sizes, and needed sample sizes. Results for ME-ICA preprocessing and the top 1,000 voxel mask are shown in Figure 8. Surprisingly, the mean BOLD-RT correlation values are not strongly affected by the number of trials (Figure 8A). Indeed, there are no significant differences in the mean values among any combination of the trial conditions (*p* > 0.2 for all; see matrix of *t*-values to the right in Figure 8A). However, the variability of the values across participants decreases strongly with a greater number of trials included (as seen by the shrinking of the 95% confidence intervals around the means with increasing numbers of trials in Figure 8A). This decrease in variability is what drives larger effect sizes at the group level (Figure 8B). When considering effect sizes at the group level, the values of Cohen’s *d* improve from 0.1466 for 5 trials up to 0.8022 for 50 trials (and 1.221 for all trials). Using bootstrap resampling to generate sampling distributions of the conditions (and of the differences of the conditions; see matrix of *z*-values to the right in Figure 8B), we find that 5 trials is significantly weaker in effect size than all other conditions (*p* < 0.0225, *q* < 0.05 for all), and All trials is significantly greater in effect size than all other conditions (*p* < 0.0140, *q* < 0.05 for all). No other combinations of trial conditions survived correction for multiple comparisons. The corresponding needed sample sizes for these effect sizes are shown in Figure 8C. With only 5 trials included (effect size of 0.1466), the expectation is that more than 360 participants would be needed to find an effect at *p* < 0.05 and 80% power. This number decreases dramatically even by 10 trials (36 participants) and is within the typical sample size of most studies in the field (approximately 25 participants) by 20–30 trials. Taken together, the results suggest that BOLD-RT correlations measured across trials, despite weak effect sizes for individual participants, can be robust and replicate when evaluated at the group level with 20–30 participants and 30 or more trials per participant.

## Discussion

In an experiment with 40 participants performing an object naming task, we have observed robust and replicable effects of task and of trial-level BOLD-RT correlations. ME-ICA denoising of multi-echo data yielded the strongest BOLD-RT correlations in our experiment (Cohen’s *d*’s > 1.0), but strong effects (Cohen’s *d*’s of 0.9–1.0) were observed even for more traditional single-echo data. The slow event-related design used here is undoubtedly an impactful choice, allowing the improved isolation of individual trial responses over more traditional rapid event-related designs—which are primarily concerned with estimating condition-level mean BOLD responses across trials. Without the ability to estimate trial-level effects, we would be restricted to calculating brain-behavior correlations across participants as in most previous studies, potentially more limited in effect sizes (estimates of 0.4–0.6, e.g., Vul et al., 2009). While the BOLD-RT correlation magnitudes for individual participants were observed to be weak (correlation effect sizes of <0.1), these values were reliably different from zero across participants—permitting much larger effect sizes when considering the group-level means. As anticipated, this group-level reliability hinged on the number of trials included, with reasonably robust effects observable with 30 or more trials included (see Chen et al., 2022, for related discussion). With all correct trials included (more than 90 trials per participant on average), we estimated that as few as 10 participants might be needed in a sample to observe replicable results at *p* < 0.05. This is not to suggest that small sample sizes will suffice for all such effects; the needed sample size for a given study will depend on the particular effect sizes involved and the desired level of significance and power.

The conclusions of Marek et al. (2022) that BWAS studies will likely require thousands of participants to detect reliable findings has predictably set off intense discussion within the field of cognitive neuroscience about whether the current state of affairs is really so dire (e.g., Bandettini et al., 2022; Gratton et al., 2022; Liu et al., 2023; Rosenberg and Finn, 2022; Botvinik-Nezer and Wager, 2022; Spisak et al., 2023; Tervo-Clemmens et al., 2023; Westlin et al., 2023; Wu et al., 2022). In the current paper, we have detailed a task-based alternative to detecting brain-behavior relationships that primarily utilizes inter-trial variability in the BOLD response and behavior rather than inter-individual variability. Taking this approach in the current study turned trial-level effect sizes similar to what Marek et al. (2022) reported (< 0.1) into group-level effect sizes that are an order of magnitude larger (1.0 or more). The savings in sample size to produce replicable findings is dramatic, going from thousands to tens. For domains of cognition that can be studied with impulse-response type tasks with fMRI (stimulus duration of approximately 1 s or less, followed by an immediate response), this type of design provides a good option for making quick progress in individual labs. It is also worth revisiting the task-based approach reviewed by Vul et al. (2009) (see also Rousselet and Pernet, 2012; Yarkoni and Braver, 2010) that utilizes inter-individual variability in task activation and behavior. If unbiased effect sizes are actually in the range of 0.4–0.6, sample sizes of 30–40 participants may be sufficient to detect replicable effects. In one of our recent studies (Gotts et al., 2021), we correlated the magnitude of repetition-related BOLD decrease (referred to as “repetition suppression”) in left frontal cortex with the magnitude of behavioral repetition priming across 60 participants. This particular analysis was conducted as a replication of prior studies (e.g., Dobbins et al., 2004: *N* = 16 participants; Horner and Henson, 2008: *N* = 18; Maccotta and Buckner, 2004: *N* = 54), and indeed, it did replicate—despite utilizing inter-individual variability in BOLD and RT differences (OLD vs. NEW stimuli) [*r*(58) = 0.367, *p* < 0.004].

We observed larger BOLD-RT correlations and group-level effect sizes using ME-ICA processing. This adds another data point in favor or utilizing multi-echo acquisitions, and using ME-ICA, in particular (see also Beckers et al., 2023; Kundu et al., 2013; Gilmore et al., 2022; Reddy et al., 2024; Steel et al., 2022). The advantage in using ME-ICA here likely results from the removal of non-BOLD variation from the total variation, improving the signal-to-noise ratio (SNR) of the retained signal and strengthening the BOLD-RT correlation. Similarly, the likely benefit of the Optimally Combined preprocessing over Single-Echo is the reduction of thermal noise that occurs when averaging across the three echoes at each TR. At a field strength of 3 Tesla with 3-mm isometric voxel resolution, thermal noise still makes up a sizable portion of the time series SNR (e.g., Triantafyllou et al., 2005), and reducing it through local averaging (either temporally for multi-echo or spatially for single-echo acquisitions) should lead to larger brain-behavior correlations as observed here. Given the reduction of thermal noise when using multi-echo acquisitions without the need for spatial averaging, an added advantage of multi-echo protocols is improved spatial localization within individual participants.

While we do not have an explicit estimate in the current study of test–retest reliability of single-trial BOLD responses, they appear to have sufficient reliability that detection of BOLD-RT correlations is possible—despite the poor reliability of individual RTs (approximately 0.17). This suggests that slow event-related designs have a notable advantage over rapid-event related designs, namely that both the mean of individual conditions can be estimated as well as the values of individual trials. As can be seen from the single-participant example in Figure 3C, the peaks of individual trial responses are visible by eye without complex statistical analyses (see also Bandettini and Cox, 2000). This is the case in the current study, even without requiring interstimulus intervals greater than or equal to the full duration of the BOLD response (approximately 16 s); the average interstimulus interval in the current study was 9.6 s (minimum 6.3 s, maximum 12.9 s). A wide range of cognitive tasks can be conducted using this range of trial timings without participants losing focus. The loss in number of stimulus presentations when compared to rapid event-related designs is also mitigated by the fact that no additional baseline periods are needed in slow event-related designs (whereas approximately 30% of each run duration in rapid event-related designs are reserved for baseline periods). A final added advantage of this type of slow event-related task design is that it can provide a way to more cleanly estimate task-based functional connectivity in fMRI in a manner that is less confounded by the local stimulus response itself. Recently, Gotts et al. (2021) in the domain of repetition priming in picture naming showed how to use trial covariation as a measure of functional connectivity separate from the mean BOLD response to the task. By eliminating the up-and-down contour of the evoked response and focusing on the peak response to each trial, one can evaluate the covariation of individual trial amplitudes across pairs of voxels or brain regions, investigating differences in functional and/or effective connectivity for different stimulus conditions (e.g., OLD vs. NEW), as well as correlating task-based connectivity measures with behavior.

The current approach is limited to investigating brain-behavior correlations using the BOLD response in fMRI. This approach has the advantage of whole-brain coverage and good spatial resolution. However, the temporal resolution is relatively poor, and the averaging inherent in the BOLD response does not allow unambiguous estimates of the underlying neural activity. Combining this approach with a method like EEG that has the temporal resolution of milliseconds (e.g., simultaneous fMRI-EEG) may help to undercover additional features of brain-behavior relationships that are not possible to study with either neuroimaging method used in isolation. The behavioral task examined here is also relatively simple. More complex tasks that are more extended in time will likely require more sophisticated analyses (for example, see Gilmore et al., 2021; Jasmin et al., 2023). In these situations, the trial-level approach may be less applicable.

How could we extend the current approach to a richer investigation of behavior? Firstly, cognitive psychology has utilized response time and accuracy as primary measures of behavioral variability in tasks since the 1970s. These measures are themselves demonstrably rich. However, one can think of alternative ways to gain further insights. For example, if one were to norm a stimulus set on a variety of behavioral measures in an independent set of participants, one could then seek trial-level correlations with the BOLD response using these “external” measures of behavior per trial. Here, we could have used lexical frequency of the object names (e.g., Kucera and Francis, 1967), familiarity, or visual complexity (e.g., Snodgrass and Vanderwart, 1980) as trial-level measures to correlate with our single-trial BOLD responses rather than the simultaneously acquired response times. The role of the naming task then would be to force participants to deeply process and engage with the stimuli such that these alternate behavioral facets might be expressed in a subset of the brain regions detected in the task overall.

## Data Availability

The raw data supporting the conclusions of this article will be made available by the authors, without undue reservation.
